# Treosulfan vs busulfan conditioning for allogeneic bmt in children with nonmalignant disease: a randomized phase 2 trial

**DOI:** 10.1038/s41409-023-02135-9

**Published:** 2023-11-04

**Authors:** Karl-Walter Sykora, Rita Beier, Ansgar Schulz, Simone Cesaro, Johann Greil, Jolanta Gozdzik, Petr Sedlacek, Peter Bader, Johannes Schulte, Marco Zecca, Franco Locatelli, Bernd Gruhn, Dirk Reinhardt, Jan Styczynski, Simona Piras, Franca Fagioli, Sonia Bonanomi, Maurizio Caniglia, Xieran Li, Joachim Baumgart, Jochen Kehne, Monika Mielcarek-Siedziuk, Krzysztof Kalwak

**Affiliations:** 1 Hannover Medical School, Ped. Haematology and Oncology, Hannover, Germany; 2 Department of Pediatrics, University Medical Center Ulm, Ulm, Germany; 3 Pediatric Hematology Oncology, Azienda Ospedaliera Universitaria Integrata, Verona, Italy; 4 University Hospital, Heidelberg, Germany; 5 Jagiellonian University Medical College, Center of Transplantation University Children’s Hospital in Cracow, Cracow, Poland; 6 University Hospital Motol, Prague, Czech Republic; 7 University Hospital Frankfurt, Frankfurt Main, Germany; 8 Charité University Hospital, Berlin, Germany; 9 Children’s Hospital San Matteo, Pavia, Italy; 10 Children’s Hospital Bambino Gesú, Rome, Italy; 11 Department of Pediatrics, Jena University Hospital, Jena, Germany; 12 University Hospital, Essen, Germany; 13 Department of Pediatric Hematology and Oncology, University Hospital, Collegium Medicum UMK, Bydgoszcz, Poland; 14 Children’s Hospital Antonio Cao, Cagliari, Italy; 15 Children’s Hospital Regina Margherita, Turin, Italy; 16 University Hospital, Monza, Italy; 17 Children’s Hospital S. Maria della Misericordia, Perugia, Italy; 18 medac GmbH, Wedel, Germany; 19 Department of Pediatric Hematology, Oncology and BMT, Wroclaw Medical University, Wroclaw, Poland

**Keywords:** Randomized controlled trials, Drug development

## Abstract

Optimal conditioning prior to allogeneic hematopoietic stem cell transplantation for children with non-malignant diseases is subject of ongoing research. This prospective, randomized, phase 2 trial compared safety and efficacy of busulfan with treosulfan based preparative regimens. Children with non-malignant diseases received fludarabine and either intravenous (IV) busulfan (4.8 to 3.2 mg/kg/day) or IV treosulfan (10, 12, or 14 g/m^2^/day). Thiotepa administration (2 × 5 mg/kg) was at the investigator’s discretion. Primary endpoint was freedom from transplantation (treatment)-related mortality (freedom from TRM), defined as death between Days -7 and +100. Overall, 101 patients (busulfan 50, treosulfan 51) with at least 12 months follow-up were analyzed. Freedom from TRM was 90.0% (95% CI: 78.2%, 96.7%) after busulfan and 100.0% (95% CI: 93.0%, 100.0%) after treosulfan. Secondary outcomes (transplantation-related mortality [12.0% versus 3.9%]) and overall survival (88.0% versus 96.1%) favored treosulfan. Graft failure was more common after treosulfan (n = 11), than after busulfan (n = 2) while all patients were rescued by second procedures except one busulfan patient. CTCAE Grade III adverse events were similar in both groups. This study confirmed treosulfan to be an excellent alternative to busulfan and can be safely used for conditioning treatment in children with non-malignant disease.

## Introduction

Allogeneic hematopoietic stem cell transplantation (HSCT) provides a curative treatment for pediatric patients affected with non-malignant diseases like primary immunodeficiencies (PID), haemoglobinopathies (HBP), bone marrow failure (BMF) syndromes, or inborn errors of metabolism (IEM) [1–7]. For these non-malignant diseases a variety of mainly chemotherapy based conditioning regimens are applied. They include cytotoxic agents as busulfan, treosulfan, cyclophosphamide, thiotepa or melphalan. Significant morbidity and mortality risks exist for children undergoing allogeneic HSCT [8, 9]. The use of reduced intensity or reduced toxicity conditioning regimens to decrease risks of conditioning-related morbidities is restricted by the need of sustained engraftment with a sufficient percentage of donor-type chimerism to ensure disease-free survival.

Treosulfan’s (L-threitol-1,4-bis-methanesulfonate) potential for myeloablative conditioning with low toxicity was first demonstrated in adults [10–13] and then in children with malignancies [14–18]. It is approved in combination with fludarabine in the EU, Switzerland, Australia, and Canada [19]. However, in essentially all non-malignant transplant indications, extensive experience already exists with treosulfan based conditioning in the form of case series [18, 20, 21], single-arm prospective studies [21–24], or retrospective registry analyses [5, 25, 26].

We prospectively compared safety and efficacy of treosulfan/fludarabine with busulfan/fludarabine myeloablative conditioning in children with non-malignant disease. The trial was conducted in accordance with the approved European pediatric investigational plan for treosulfan (PIP; EMEA-000883-PIP01-10) including a pharmacokinetic (PK) sub-study.

## Materials and methods

### Study design

A prospective, randomized (1:1), open-label, multicenter, active-controlled, parallel-group phase 2 clinical trial (MC-FludT.16/NM) was conducted across 4 European countries between April 2015 to June 2021. Each treatment arm also administered fludarabine whereas thiotepa could be added for intensification of the regimen at the treating physicians’ discretion before randomization. Pharmacokinetic analyses on treosulfan were conducted to contribute to a final population pharmacokinetics (Pop-PK) model. The study was conducted in accordance with the ethical principles of the Declaration of Helsinki and the applicable national laws of the participating countries (Czech Republic, Germany, Italy, Poland). All patients and/or their parents/legal guardians provided written consent prior to the participation in the study.

Randomization (1:1 ratio using a permuted block technique) for either treosulfan or busulfan was performed centrally by the sponsor’s clinical research department using a computer-generated randomization list and was stratified by the 2 regimens. Treosulfan was administered IV over 2 hours consecutively on Day -6, -5, and -4. Based on an initially evaluated Pop-PK model, the individual total dose of treosulfan was adapted on the actual body surface area (BSA) [27]. Accordingly, 10, 12, or 14 g/m² treosulfan was administered to patients with BSA ≤ 0.5 m², >0.5 to ≤1.0 m², >1.0 m², respectively. Busulfan was given IV on 4 consecutive days (4.8 to 3.2 mg/kg/day on Days -7, -6, -5, and -4, according to the actual body weight) and thiotepa (2 × 5 mg/kg on Day -2). All patients were observed until Day +100 after HSCT for acute toxicity and freedom from transplantation (treatment)-related mortality. The follow-up was continued for each patient until at least 12 months after HSCT.

Further information for actual administration of the preparative regimens is provided (Supplementary Section 1.3).

### Study participants

Pediatric patients 28 days to less than 18 years of age with nonmalignant disease including IEM, PID, HBP, and BMF were eligible. Only patients with an indication for a first allogeneic HSCTs were enrolled if a matched sibling donor, matched family donor, matched unrelated donor, or umbilical cord blood was available. Lansky Performance Scores or Karnofsky Performance Scores for those ≥16 years of age had to be at least 70%. Main exclusion criteria included obese pediatric patients with body mass index > 30 kg/m^2^, patients with Fanconi anemia and other chromosomal breakage or radiosensitivity disorders, trisomy 21, and Dyskeratosis Congenita.

### Study objectives

The primary objective was to compare freedom from transplantation (treatment)-related mortality (freedom from TRM), defined as death from any transplantation (treatment)-related cause from start of conditioning treatment (Day -7) until Day +100 after allogeneic HSCT. Toxicity was documented using the Common Terminology Criteria for Adverse Events (CTCAE, version 4.03) until Day +100 and serious adverse reactions until the end of follow-up.

Comparative exploratory analyses also included engraftment, primary or secondary graft failure, complete ( ≥ 95%) or mixed ( ≥ 20%) donor-type chimerism, transplantation-related mortality (TRM), overall survival (OS), acute [28, 29] and chronic [30] graft versus host disease (GVHD), and GVHD-free survival as previously described [16]. A more detailed description of the secondary endpoints is provided (Supplementary Section 1.1 and 1.2).

### Statistical analysis plan

The trial was not powered for confirmatory statistical testing of any pre-specified hypotheses. Following the approved PIP, at least 100 evaluable children had to be enrolled. Descriptive statistics including 95% confidence intervals (CI) was applied to summarize all endpoints, including baseline characteristics and covariates used in multivariate analyses. Three (2.9%) umbilical cord blood transplanted patients were included in the matched unrelated donor (2) and matched family donor (1) subgroup (Supplementary Table 3). The following analyses were done to compare endpoints between treatment arms. Fisher’s exact test was used for rate of hepatic sinusoidal obstruction syndrome. Freedom from TRM, complete and mixed donor-type chimerism was analyzed using Cochran-Mantel-Haenszel tests. Duration of neutropenia and leukopenia was evaluated with Wilcoxon-Mann-Whitney tests.

All time-to-event endpoints were measured from the day of HSCT (except for chronic GVHD [cGVHD] from 100 days after HSCT) to the event or competing event (if applicable). The probability of event over time for freedom from TRM, TRM, OS, and GVHD-free survival was estimated by Kaplan-Meier estimator, and for engraftment, primary and secondary graft failure until 12 months after HSCT, incidence of acute GVHD (aGVHD) and cGVHD was estimated by cumulative incidence functions due to competing risks. For comparisons, Pepe-Mori tests for engraftment were performed. Cox models for freedom from TRM, TRM, OS, and GVHD-free survival, and Fine and Gray models for engraftment, graft failure, and incidence of aGVHD and cGVHD were applied to adjust for covariates in multivariate analyses. The following covariates are additionally considered to examine efficacy and safety in prespecified subgroups or in multivariate analyses (disease groups, age group, donor type, thiotepa, and serotherapy). All analyses were predefined and SAS software (Version 9.4) was used.

### Pharmacokinetic assessment (treosulfan)

Patients of the PIP pre-specified age groups were included in the PK sub-study for both pediatric allogeneic HSCT trials MC-FludT.16/NM and MC-FludT.17/M [16]. Blood samples were taken by limited sampling procedure as previously described [16]. The non-compartmental analysis was applied based on the individual plasma concentration-time- data. The following pharmacokinetic parameters were determined as previously described [16]: maximum observed concentration, time to reach maximum plasma concentration, area under the time-concentration curve or from time zero to infinity, apparent terminal elimination half-life, clearance, and volume of distribution. PK parameters were also stratified by BSA. Further details of bioanalytical methods and the model-based PK parameter calculation have been previously described [16, 31].

Pharmacokinetic analyses used the Phoenix™ WinNonlin^®^ (version 6.2.1). Non-compartmental analysis model 202 (constant infusion input, plasma data) was applied.

## Results

### Patient characteristics

**Fig. 1:**
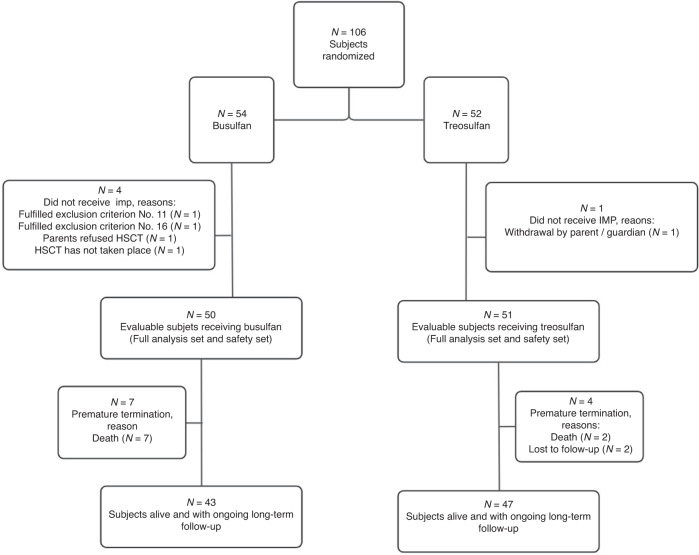
Consort diagramm. Patient disposition, for In- and Exclusion Criteria see Supplementary Information (1.4).

A total of 106 patients were randomized of which 101 patients received the study drug, underwent allogeneic HSCT, and were included in the efficacy and safety analyses (Fig. 1). More than half of the patients were male (66.3%) and mean age of all patients was 6.0 ( ± 5.3) years. Underlying diseases were PID (n = 53), HBP (n = 35), BMF (n = 11), and IEM (n = 7) (Table 1). Among patients with HBPs, only 5 (38.5%) beta-thalssaemia patients were in the busulfan arm and 16 (76.2%) in the treosulfan arm. In the busulfan arm 72.0% of patients had a Lansky Performance Score of 100% compared to 82.4% in the treosulfan arm (Table 1). Depending on their individual BSA, patients received treosulfan at a dose of 10 g/m^2^/day (17.3%), 12 g/m^2^/day (61.5%), or 14 g/m^2^/day (21.2%) on three consecutive days.

**Table 1:** Baseline demographics and disease characteristics.

	Busulfan (N = 50)	Treosulfan (N = 51)
Gender, n (%)		
Female	19 (38.0)	15 (29.4)
Male	31 (62.0)	36 (70.6)
Age, years; mean (SD)	6.0 (5.3)	5.0 (4.4)
ICH age group, n (%)		
28 days to 23 months	14 (28.0)	14 (27.5)
2 to 11 years	26 (52.0)	31 (60.8)
12 to 17 years	10 (20.0)	6 (11.8)
Race, n (%)		
White	43 (86.0)	41 (80.4)
Black or African American	2 (4.0)	2 (3.9)
Asian	1 (2.0)	6 (11.8)
Other	4 (8.0)	2 (3.9)
Weight, kg; mean (SD)	23.6 (15.9)	19.7 (11.3)
BMI, kg/m2; mean (SD)	16.97 (3.07)	16.77 (2.20)
Body surface area, m2; mean (SD)	0.836 (0.396)	0.746 (0.297)
Median time between diagnosis and HSCT (months)		
Primary immunodeficiencies	7.85	8.38
Inborn error of metabolism	5.75	6.34
Haemoglobinopathies	89.91	67.61
Bone marrow failure syndromes	37.62	27.24
Disease groups, n (%)		
Primary immunodeficiency	28 (56.0)	23 (45.1)
Inborn error of metabolism	4 (8.0)	2 (3.9)
Haemoglobinopathy	13 (26.0)	21 (41.2)
Beta-thalassemia major	5 (10.0)	16 (31.4)
Sickle cell disease	8 (16.0)	5 (9.8)
Bone marrow failure syndrome	5 (10.0)	5 (9.8)
Donor type, n (%)		
MRD	17 (34.0)	14 (27.5)
MUD	33 (66.0)	37 (72.5)
Applied performance score*, n (%)		
Lansky performance score	48 (96.0)	50 (98.0)
70	2 (4.0)	1 (2.0)
80	1 (2.0)	0 (0.0)
90	9 (18.0)	7 (13.7)
100	36 (72.0)	42 (82.4)
Karnofsky performance score	2 (4.0)	1 (2.0)
100	2 (4.0)	1 (2.0)
Thiotepa, n (%)		
No	8 (16.0)	8 (15.7)
Yes	42 (84.0)	43 (84.3)
Serotherapy, n (%)		
No	18 (36.0)	13 (25.5)
Yes	32 (64.0)	38 (74.5)

*Lansky score if age <16 years at registration; Karnofsky score if age ≥16 years at registration.

*BMI* body mass index, *HSCT* hematopoietic stem cell transplantation, *ICH* International Council for Harmonization of Technical Requirements for Pharmaceuticals for Human Use, *MRD* matched related donor, *MUD* matched unrelated donor, *SD* standard deviation.

### Efficacy results

The incidence of freedom from TRM until Day +100 was 90.0% (95% CI: 78.2%, 96.7%) and 100.0% (95% CI: 93.0%, 100.0%) in the busulfan and treosulfan arm (difference of incidences –10.0% [95% CI: –21.8%, –2.0%]; *P* = 0.0528) (Table 2). Until Day +100, five patients (10.0%) had died from transplantation or a treatment-related cause in the busulfan arm. No death was reported in the treosulfan arm. A beneficial outcome for treosulfan regarding the primary endpoint was evident across all predefined subgroups including disease group, age group, donor type, thiotepa and serotherapy (Supplementary Fig. 3).

**Table 2:** Freedom from Transplantation (treatment)-related Mortality and Secondary Outcomes (FAS).

	Busulfan	Treosulfan
	(N = 50)	(N = 51)
Freedom from transplantation (treatment)-related mortality until Day + 100		
Patients without event, n (%)	45 (90.0%)	51 (100.0%)
Incidence, % (95% CI)	90.0 (78.2, 96.7)	100.0 (93.0, 100.0)
Difference in incidences, % (95% CI)	–10.0 (–21.8, –2.0)	
P *†	0.0528	
TRM		
Patients with event, n (%)	7 (14.0)	2 (3.9)
TRM at 12 months‡, % (95% CI)	12.0 (5.6, 24.8)	3.9 (1.0, 14.8)
Hazard ratio (Treosulfan/Busulfan)§ (95% CI)	0.29 (0.08, 1.09)	
P §	0.1244	
OS		
Patients without event, n (%)	43 (86.0)	49 (96.1)
OS at 12 months‡, % (95% CI)	88.0 (75.2, 94.4)	96.1 (85.2, 99.0)
Hazard ratio (Treosulfan/Busulfan)§ (95% CI)	0.29 (0.06, 1.41)	
P §	0.1244	
Engraftment		
Reconstitution of granulopoiesis, n (%)		
Patients with event	36 (72.0)	40 (78.4)
Patients without event (censored) or with competing event	14 (28.0)	11 (21.6)
Censored	2 (4.0)	2 (3.9%)
Death | |	0 (0.0%)	0 (0.0%)
Rescue therapy | |	12 (24.0%)	9 (17.6%)
Conditional cumulative incidence at 28 days, % (95% CI)	88.5 (75.9, 100.0)	81.0 (65.8, 96.1)
Maximum conditional cumulative incidence reached, % (95% CI)	100.0 (93.0, 100.0)	97.3 (87.0, 100.0)
P	0.0521	
Neutropenia		
Yes#	50 (100.0%)	51 (100.0%)
Duration of neutropenia, days**		
n	48	49
Median (Q1, Q3)	14.5 (10.0, 21.0)	20.0 (15.0, 25.0)
P ††	0.0108	
Reconstitution of leukopoiesis, n (%)		
Patients with event	36 (72.0)	40 (78.4)
Patients without event (censored) or with competing event	14 (28.0)	11 (21.6)
Censored	2 (4.0)	2 (3.9)
Death | |	0 (0.0)	0 (0.0)
Rescue therapy | |	12 (24.0)	9 (17.6)
Conditional cumulative incidence at 28 days, % (95% CI)	88.5 (75.7, 100.0)	90.5 (82.2, 98.8)
Maximum conditional cumulative incidence reached, % (95% CI)	100.0 (93.0, 100.0)	96.8 (85.3, 100.0)
P	0.2469	
Leukopenia		
Yes‡‡	50 (100.0%)	51 (100.0%)
Duration of leukopenia, days a		
n	48	49
Median (Q1, Q3)	14.5 (12.0, 20.0)	19.0 (16.0, 22.0)
P ††	0.0087	
Reconstitution of thrombopoiesis > 20 × 109/L, n (%)		
Patients with event	35 (70.0)	40 (78.4)
Patients without event (censored) or with competing event	15 (30.0)	11 (21.6)
Censored	3 (6.0)	2 (3.9)
Death | |	0 (0.0)	0 (0.0)
Rescue therapy | |	12 (24.0)	9 (17.6)
Conditional cumulative incidence at 28 days, % (95% CI)	77.6 (61.4, 93.9)	85.7 (75.6, 95.8)
Maximum conditional cumulative incidence reached, % (95% CI)	96.8 (84.6, 100.0)	100.0 (92.7, 100.0)
P	0.8595	
Graft failure		
Patients with event b, n (%)	2 (4.0)	11 (21.6)
Primary graft failure	2 (4.0)	2 (3.9)
Secondary graft failure	0 (0.0)	9 (18.4)
Cumulative incidence at 12 months, % (95% CI)	4.0 (0.0, 9.4)	15.8 (5.8, 25.9)
Hazard ratio (Treosulfan/Busulfan) c (95% CI)	5.48 (1.11, 27.03)	
P c	0.0366	
Incidence of complete donor type chimerism until Month 12		
Patients at risk at Day +28 d	50	51
Patients with complete chimerism, n (%)	41 (82.0)	43 (84.3)
Patients without information, n (%)	0 (0.0)	1 (2.0)
Odds ratio e* (95% CI)	1.5824 (0.51, 4.89)	
P e*†	0.425	
Patients at risk at Day +100 d	46	51
Patients with complete chimerism, n (%)	39 (84.8)	34 (66.7)
Patients without information, n (%)	2 (4.3)	3 (5.9)
Odds ratio e* (95% CI)	0.3972 (0.12, 1.28)	
P e*†	0.1196	
Patients at risk at Month 12 d	43	49
Patients with complete chimerism, n (%)	33 (76.7)	24 (49.0)
Patients without information, n (%)	1 (2.3)	11 (22.4)
Odds ratio e* (95% CI)	0.5429 (0.20, 1.51)	
P e*†	0.2445	
Incidence of mixed donor type chimerism (with at least 20% chimerism) until Month 12		
Patients at risk at Day +28 d	50	51
Patients with ≥20% chimerism, n (%)	49 (98.0)	48 (94.1)
Patients without information, n (%)	0 (0.0)	1 (2.0)
Odds ratio e* (95% CI)	0.3041 (0.02, 4.32)	
P e*†	0.3679	
Patients at risk at Day +100 d	46	51
Patients with ≥ 20% chimerism, n (%)	44 (95.7)	46 (90.2)
Patients without information, n (%)	2 (4.3)	3 (5.9)
Odds ratio e* (95% CI)	<0.0001 (NE)	
P e*†	0.3173	
Patients at risk at Month 12 d	43	49
Patients with ≥ 20% chimerism, n (%)	42 (97.7%)	37 (75.5%)
Patients without information, n (%)	1 (2.3)	11 (22.4)
Odds ratio e* (95% CI)	<0.0001 (NE)	
P e*†	0.4142	
GVHD-free survival		
Patients with event, n (%)	15 (30.0)	8 (15.7)
Death | |	4 (8.0)	0 (0.0)
Acute GVHD of at least Grade III	4 (8.0)	7 (13.7)
Moderate/severe chronic GVHD	7 (14.0)	1 (2.0)
GVHD-free survival at 12 months‡, % (95% CI)	69.4 (54.4, 80.3)	82.9 (68.7, 91.1)
Hazard Ratio (Treosulfan/Busulfan)§ (95% CI)	0.58 (0.24, 1.38)	
P §	0.2178	

*Adjusted for thiotepa and disease.

†Stratified Cochran-Mantel-Haenszel test.

‡Based on Kaplan-Meier estimates.

§Adjusted for thiotepa and disease as factors using Cox regression model.

||Only if this event occurred first.

¶Based on Pepe-Mori test.

#Neutrophilic granulocytes ≤ 0.5 g/L at least once between Day -7 and Day +28.

∗∗First date with neutropenia until date of engraftment (patients at risk = patients with neutropenia and neutrophilic granulopoiesis).

††Based on the Wilcoxon-Mann-Whitney test.

‡‡Leukocytes ≤ 1 g/L at least once between Day -7 and Day +28.

aFirst date with leukopenia until date of engraftment (patients at risk = patients with leukopenia and leukopoiesis).

bRate of primary/secondary graft failure calculated as number of patients with graft failure by the number of patients at risk.

-At risk for primary graft failure: Patients with HSCT.

-At risk for secondary graft failure: Patients whose neutrophilic granulocytes engrafted after HSCT or were never below the required level.

cAdjusted for thiotepa and disease as factors using Fine and Gray model.

dPatients are at risk if they have an examination at the Day +28, Day +100, Month 12 or if they have survived day +30, +107, +379, respectively.

eMissing values are excluded for odds ratio calculation and tests.

CI, confidence interval; FAS, full analysis set; GVHD, Graft‑versus-host disease; N, total number of patients; NE, not estimated; OS, overall survival; Q1, first quartile; Q3, third quartile; TRM, transplantation related mortality.

**Fig. 2:**
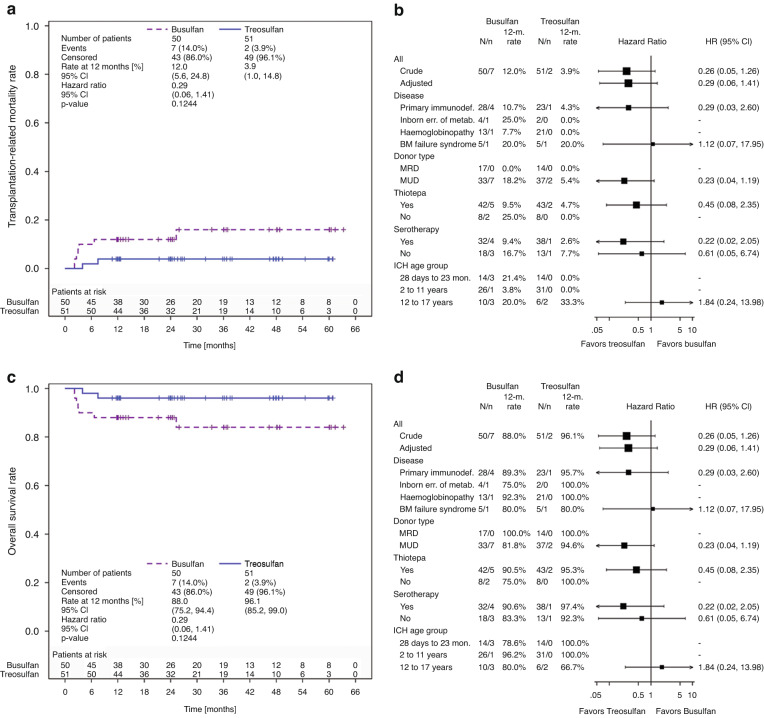
Kaplan-Meier curves and forest plots for transplantation-related mortality and overall survival. All Death Events in Overall Survival are Attributed to Events of Transplantation-related Mortality **a** Kaplan-Meier estimate of transplantation-related mortality of children with non-malignant disease randomized to treosulfan or busulfan based conditioning prior to allogeneic transplantation (FAS). **b** Forest plot for transplantation-related mortality displaying 12-month rates by subgroups (FAS) **c** Kaplan-Meier estimate of overall survival of children with non-malignant disease randomized to treosulfan or busulfan based conditioning prior to allogeneic transplantation (FAS). **d** Forest plot for overall survival displaying 12-month rates by subgroups (FAS).

The Kaplan-Meier estimate of TRM at 12 months was 12.0% (95% CI: 5.6%, 24.8%) and 3.9% (95% CI: 1.0%, 14.8%) in the busulfan and treosulfan arm (HR: 0.29 [95% CI: 0.06, 1.41]). Estimate of TRM at 12 months in the disease subgroup HBPs was 7.7% for busulfan and 0% for treosulfan. After a median follow-up of 25 months (busulfan range: 11.7-63.3 months; treosulfan range: 10.7-60.9 months) the 12-month estimate of OS was 88.0% (95% CI: 75.2%, 94.4%) in the busulfan arm versus 96.1% (95% CI: 85.2%, 99.0%) in the treosulfan arm (HR: 0.29 [95% CI: 0.06, 1.41]; Fig. 2, Table 2). OS estimate in the subgroup of HBPs was 92.3% for busulfan and 100% for treosulfan. Infection-related deaths were more frequently observed in the busulfan arm (10.0%) than in the treosulfan arm (2.0%) (Supplementary Table 2).

The conditional cumulative incidence of neutrophil engraftment was comparable between the treatment arms (busulfan: 100.0% [95% CI: 93.0%, 100.0%] and treosulfan: 97.3% [95% CI: 87.0%, 100.0%]) (Table 2). The median duration of CTCAE Grade IV neutropenia was significantly shorter in the busulfan arm (busulfan: 14.5 days [interquartile range {IQR}: 10.0, 21.0]) compared to treosulfan (20.0 days [IQR: 12.0, 22.0], *P* = 0.0108). Similar results were seen for the median duration of CTCAE Grade IV leukopenia (busulfan: 14.5 days [IQR: 12.0, 20.0] and treosulfan: 19.0 days [IQR: 13.0, 21.0], *P* = 0.0087).

Primary graft failure was noted in 2 patients each in the busulfan arm (4.0%) and the treosulfan arm (3.9%). However, none of the patients (0%) in the busulfan arm experienced a secondary graft failure as compared to 9 patients (18.4%) in the treosulfan arm (Table 2 and Supplementary Table 3). Overall, cumulative incidences of primary and secondary graft failures at 12 months were 4.0% (95% CI: 0.0%, 9.4%) versus 15.8% (95% CI: 5.8%, 25.9%) respectively (*P* = 0.0366) (Supplementary Fig. 4). Cumulative incidence of graft failures in the subgroup of HBPs reached 0% after busulfan and 9.5% in the treosulfan treatment group.

The fraction of patients with complete donor-type chimerism decreased between Day +28 and Month 12 in both treatment arms (busulfan: from 82.0% to 76.7%; treosulfan: from 84.3% to 49.0%) (Table 2). The odds ratio at Month 12 was 0.5429 (95% CI: 0.20, 1.51). Incidence of complete donor-type chimerism at month 12 in the subgroup of HBPs was 66.7% after busulfan and 42.9% after treosulfan. The fraction of all patients with mixed donor-type chimerism of ≥20% between Day +28 and Month 12 remained nearly unchanged in the busulfan arm (from 98.0 to 97.7) whereas it declined in the treosulfan arm from 94.1% to 75.5%. Two patients (4.0%) in the busulfan arm and 5 patients (9.8%) in the treosulfan arm received donor lymphocyte infusions.

Acute GVHD of at least Grade III was noted in 4 patients (8.0%) in the busulfan arm as compared to 7 patients (13.7%) after treosulfan (Table 3). However, moderate/severe cGVHD was observed more frequently in patients treated with busulfan (7 [14.0%]) compared to treosulfan (1 [2.0%]). Fifteen patients (30.0%) in the busulfan arm and 8 patients (15.7%) in the treosulfan arm experienced either death, aGVHD of at least Grade III, or moderate / severe cGVHD. The corresponding Kaplan-Meier estimate of GVHD-free survival at 12 months was 69.4% (95% CI: 54.4%, 80.3%) in the busulfan arm and 82.9% (95% CI: 68.7%, 91.1%) in the treosulfan arm (HR: 0.58 [95% CI: 0.24, 1.38]) (Table 2 and Supplementary Fig. 1). Chronic GVHD-free survival at Month 12 was 69.4% (95% CI: 54.4%, 80.3%) after busulfan and 89.3% (95% CI: 76.2%, 95.4%) after treosulfan (difference *P* = 0.0308), being statistically significant in favor of treosulfan (Supplementary Fig. 2).

**Table 3:** Graft-versus-host-disease and treatment-emergent adverse events (FAS).

	Busulfan	Treosulfan
	(N = 50)	(N = 51)
Acute GVHD		
GVHD Grade I-IV		
Patients with event, n (%)	21 (42.0)	28 (54.9)
Cumulative incidence at 100 days, % (95% CI)	42.0 (28.3, 55.7)	54.9 (41.2, 68.6)
Hazard ratio (treosulfan/busulfan)*, % (95% CI)	1.65 (0.93, 2.93)	
P *	0.0889	
GVHD Grade III-IV		
Patients with event, n (%)	4 (8.0)	7 (13.7)
Cumulative incidence at 100 days, % (95% CI)	8.0 (0.5, 15.5)	13.7 (4.3, 23.2)
Hazard ratio (treosulfan/busulfan)*, % (95% CI)	1.63 (0.45, 5.92)	
P *	0.4598	
Chronic GVHD		
Moderate/Severe†	44	47
Patients with event, n (%)	10 (22.7)	5 (10.6)
Cumulative incidence at 24 months, % (95% CI)	22.7 (10.3, 35.1)	10.6 (1.8, 19.5)
Hazard ratio (treosulfan/busulfan)*, % (95% CI)	0.46 (0.15, 1.37)	
P *	0.1611	
Treatment-emergent adverse events		
Subjects with any adverse event, n (%)	48 (96.0)	49 (96.1)
P §	1.0000	
Subjects with AEs of at least CTCAE Grade III, n (%)	41 (82.0)	41 (80.4)
P §	1.0000	
CTCAE Grades I/II, n (%)	7 (14.0)	8 (15.7)
P §	1.0000	
CTCAE Grade III, n (%)	30 (60.0)	34 (66.7)
P §	0.5393	
CTCAE Grade IV, n (%)	8 (16.0)	7 (13.7)
P §	0.7862	
CTCAE Grade V, n (%)	3 (6.0)	0 (0.0)
P §	0.1176	
Serious adverse events, n (%)	16 (32.0)	18 (35.3)
P §	0.8338	

* Adjusted for thiotepa and disease as factors using Fine and Gray model.

† Patients are at risk if they have survived 100 days after end of HSCT without graft failure.

§ Fisher’s exact test.

*CI* confidence interval, *CTCAE* Common Terminology Criteria for Adverse Events, *FAS* full analysis set, *GVHD* Graft-versus-host disease, *N* total number of patients.

### Pharmacokinetic results

Due to the PIP requirements, pharmacokinetic analyses included this trial and the simultaneously performed trial for malignant hematological diseases (MC-FludT.17/M [16]). Treatment with 10, 12, or 14 g/m² treosulfan per day resulted in comparable mean maximum observed concentration and AUC values of treosulfan in plasma. A trend for increase of treosulfan exposure in the higher BSA categories was observed (Table 4).

**Table 4:** Pharmacokinetic Results of Treosulfan by BSA Group: Pooled Analysis of MC-FludT.16/NM and MC-FludT.17/M.

PK of Treosulfan corrected (mean ± SD, tmax: median [range])	BSA Group ≤ 0.5 m² (10 g/m² dose group)	BSA Group ≤ 0.5 - ≤ 1.0 m² (12 g/m² dose group)
N	15*	37†
Cmax, µg/mL	608 ± 209	662 ± 286
tmax, h	2.08 (2.00-2.50)	2.02 (2.00-2.42)
AUClast, μg.h/mL	1551 ± 474	1629 ± 402
AUC ∞ , μg.h/mL	1570 ± 482	1672 ± 401
t1/2term, h	1.27 ± 0.178	1.40 ± 0.173
CL, L/h	3.44 ± 2.78	5.32 ± 1.46
Vd, L	6.22 ± 5.26	10.7 ± 3.68

*N = 16 for t1/2term.

†N = 38 for Cmax, tmax and AUClast.

*AUC* area under the time-concentration curve, *AUC*∞, AUC from time 0 to infinite time, *AUClast* AUC from time 0 to the time of the last measurable plasma concentration, *BSA* body surface area, *CL* total clearance, *Cmax* maximum plasma concentration, *N* total number of patients, *PK* pharmacokinetic, *SD* standard deviation, *t1/2term* apparent terminal elimination half-life, *tmax* time to reach maximum plasma concentration, *Vd* volume of distribution.

### Safety

The incidences of total treatment-emergent adverse events and treatment-emergent serious adverse events were similar in the two treatment arms (Table 3). Most common treatment-emergent adverse events were oral mucositis (busulfan: 80.0%; treosulfan: 70.6%), fever (busulfan: 72.0%; treosulfan: 70.6%) and vomiting (busulfan: 64.0%; treosulfan: 66.7%) (Supplementary Table 1). The incidence of hepatic sinusoidal obstruction syndrome was higher in the busulfan arm (all grades: busulfan: 10.0%, treosulfan: 2.0%, *P* = 0.1120; ≥ Grade III according to Jones: busulfan 4.0%, treosulfan 0.0%, *P* = 0.2426). No unknown risks were identified in the trial.

Nine patients (8.9%) died until data cut-off; 7 of 50 patients (14.0%) in the busulfan arm and 2 of 51 patients (3.9%) in the treosulfan arm. All deaths were transplantation related. In both arms, most common causes were infection and GVHD associated multiple organ failure (Supplementary Table 2).

## Discussion

In this study, treosulfan-based conditioning showed a clinically meaningful trend towards improved freedom from TRM on Day +100 as well as reduced TRM at 12 months after transplantation. Also, OS and GvHD-free survival were increased, when compared to busulfan-based conditioning treatment. However, incidence of complete donor-type chimerism declined over time and an increased risk of secondary graft failure was observed after treosulfan. Accordingly, more treosulfan than busulfan treated children received second transplant procedures, donor lymphocyte infusions or stem cell boosts. Finally, all 9 patients who experienced secondary graft failure in the treosulfan arm were rescued and survived.

Meanwhile, there already is ample published clinical experience with treosulfan-based conditioning in pediatric HSCT [1, 3, 5, 21, 22, 24–26, 32–39]. Accordingly, in its guideline for HSCT for inborn errors of immunity, the EBMT Inborn Error Working Party offers treosulfan-based alternatives for conditioning treatment [40].

However, the question of the optimal preparative regimen for a patient with a particular non-malignant disease is usually answered by retrospective registry analyses of populations with a single non-malignant condition. Due to the given heterogeneity of such rare diseases the conduct of prospectively randomized trials for single specific syndromes is not considered feasible. Nevertheless, most recently published, large retrospective analyses are in line with the findings reported in our prospective study.

Albert et al. [26] analyzed 197 patients with Wiskott-Aldrich Syndrome. The 3-year OS was 88.7% and cGVHD-free survival (events include death, graft failure, and severe cGVHD) was 81.7%. OS and cGVHD-free survival were not significantly affected by the conditioning regimen (busulfan vs treosulfan-based). Patients receiving a treosulfan-based conditioning had a higher incidence of graft failure and mixed donor chimerism and more frequently underwent second procedures. The overall cumulative incidence of primary and secondary graft failure was 8.3% at 3 years. It was higher in the treosulfan (14.3%) than in the busulfan (2.9%) group, comparable to our results.

Chiesa et al. [2] retrospectively analyzed 635 children and 77 adults with chronic granulomatous disease. In this disease, the preparative regimen (busulfan vs. treosulfan) did not influence OS or event-free survival. However, univariate analysis revealed a significant impact of conditioning regimen on the overall rate of graft failures at 3 years with 10% after the treosulfan/fludarabine/thiotepa, 13% after busulfan/fludarabine, 22% after treosulfan/fludarabine and only 3% after busulfan/cyclophosphamide.

For beta-thalassemia major, Lüftinger et al. [25] performed a retrospective EBMT analysis of 772 patients, 410 of whom received busulfan/fludarabine and 362 treosulfan/fludarabine based conditioning. Two-year OS was 92.7% (95% CI: 89.3%, 95.1%) after busulfan and 94.7% (95% CI: 91.7%, 96.6%) after treosulfan. The incidence of second HSCT procedure at 2 years was 4.6% in the busulfan vs. 9.0% in the treosulfan group, representing a significant difference in the multivariate analysis. There were high cure rates in both arms of the study.

In summary, these retrospective analyses suggest that outcome differences between treosulfan or busulfan based conditioning regimens partly depend on the specific disease entity. The results of our prospective randomized study are in line with these observations regarding an improved survival, lower toxicity and cGVHD incidence, but a potentially higher rate of mixed chimerism and graft failure after treosulfan-based conditioning. For instance, in our subgroup of 21 patients with beta-thalassemia major 0 out of 5 and 3 out of 16 patients experienced a graft failure after treatment with busulfan or treosulfan, respectively. However, 100% versus 93.8% engrafted and survived at least 12 months after transplant. In our small subgroup of 13 patients with chronic granulomatous disease (CGD) 0 out of 7 and 2 out of 6 patients experienced a graft failure after treatment with busulfan or treosulfan, respectively. Finally, 6 out of 7 versus 4 out of 6 patients engrafted and survived at least 12 months after transplant. As discussed below, the patient numbers with a disease-specific indication within our trial are too small for any firm safety or efficacy conclusion. Further well-designed comparative disease-specific real world data analyses are, therefore, highly warranted as referenced above.

The PK sub-study on treosulfan included in our trials MC-FludT.16/NM and MC-FludT.17/M applied a BSA-adapted dose calculation. This was based on a Pop-PK model aiming at a comparative treosulfan exposure to all pediatric age (BSA) groups starting at 4 weeks of age [27]. Noncompartmental analysis revealed that the BSA-adapted dosing resulted in comparable exposure through the different BSA categories (Table 4). Meanwhile, several Pop-PK models have been published based on pediatric treosulfan PK data collected by various groups [41–49]. All models revealed the need for adaptation of treosulfan dose in children of less than 1 or 2 years of age. However, individualized dosing based on therapeutic drug monitoring has so far not been shown to be superior to BSA adapted dosing [50].

Despite the beneficial survival results of treosulfan based conditioning therapy as suggested by our prospective comparative trial, several limitations exist. Heterogeneity of the non-malignant transplant indications and the limited sample size affect treatment arm comparability. Randomized allocation was not stratified for underlying disease and resulted in an increased number of beta-thalassemia major in the treosulfan vs. the busulfan arm. Also, the overall study population consisted primarily of patients with PIDs and HBPs while IEMs and BMFs were underrepresented. The inclusion of patients with specific disease entities and selection of the conditioning intensity was at the investigators’ discretion. This resulted in 84% patients received the intensified treatment with thiotepa. Moreover, inclusion and exclusion criteria limited study recruitment by age, weight, body surface area, and organ function. For patients outside of these criteria, e.g., with obesity, anorexia or limited organ function the risk estimates may differ and potentially favor treosulfan. Patient numbers were too small for any potential analysis of conditioning drug exposure in subgroups.

Treating physicians may prefer treosulfan over busulfan in patients with increased risk of TRM related to e.g., concomitant infections or pre-existing organ dysfunction. Although secondary graft failures were more common in the treosulfan group, these patients were rescued by second procedures. Moreover, there is strong evidence suggesting a reduced risk for impairment of gonadal function, acute and chronic GVHD, and other early and late adverse effects after treosulfan based conditioning [51–56]. In summary, our study provides important additional evidence enabling physicians to choose the most appropriate conditioning regimen for children with non-malignant transplant indications.

### Supplementary information

Supplemental Data

## Data Availability

The datasets generated during and/or analysed during the current study are not publicly available.
