# Supplementary material for: Treosulfan vs busulfan conditioning for allogeneic bmt in children with nonmalignant disease: a randomized phase 2 trial
Source: Bone Marrow Transplant. 2023 Nov 4;59(1):107–16. doi: 10.1038/s41409-023-02135-9 (PMC10781637; doi:10.1038/s41409-023-02135-9)
Supplement: Supplementary file 1 — Supplemental Data [file 41409_2023_2135_MOESM1_ESM.docx]

SUPPLEMENTAL DATA

(Sykora et al. Manuscript MC-FludT.16/NM)

This supplemental data contains definitions of secondary objectives and endpoints, definitions of exploratory comparative analyses, and information for parenteral high dose administration of treosulfan treatment in practice, also in- and exclusion criteria are provided. This supplement also includes supporting supplemental tables and figures that are referred in the manuscript.

Table of Contents

[Table of Contents 2](#_Toc143076432)

[List of Supplemental Tables 3](#_Toc143076433)

[List of Supplemental Figures 4](#_Toc143076434)

[1 Supplemental Information 5](#_Toc143076435)

[1.1 Supplemental Information: Secondary Objectives 5](#_Toc143076436)

[1.2 Definitions of Exploratory Comparative Analyses 5](#_Toc143076437)

[1.3 Supplemental Information for Parenteral High Dose Administration of Treosulfan Treatment in Practice 6](#_Toc143076438)

[1.4 Inclusion and Exclusion criteria according to the clinical trial protocol 8](#_Toc143076439)

[2 Supplemental Tables 12](#_Toc143076440)

[3 Supplemental Figures 15](#_Toc143076441)

List of Supplemental Tables

[Supplemental Table 1: Frequency of Subjects with Treatment-emergent Adverse Events of at least CTCAE Grade III by System Organ Class and Preferred Term Occurring in at least 10% of Subjects in Either Treatment Arm 12](#_Toc141804568)

[Supplemental Table 2: Summary of Detailed Causes of Deaths 13](#_Toc141804569)

[Supplemental Table 3: All Patients with Graft Failure and Their Survival Status 14](#_Toc141804570)

List of Supplemental Figures

[Supplemental Figure 1: Kaplan-Meier Estimates of GVHD-free Survival 15](#_Toc141804571)

[Supplemental Figure 2: Kaplan-Meier Estimates of Chronic GvHD-free Survival 16](#_Toc141804572)

[Supplemental Figure 3: Forest Plot for Freedom from Transplantation (Treatment)-related Mortality Until Day +100 By Subgroups 17](#_Toc141804573)

[Supplemental Figure 4: Cumulative Incidence of Graft Failure 18](#_Toc141804574)

# Supplemental Information

## Supplemental Information: Secondary Objectives

The secondary objectives of trial MC-FludT.16/NM were as following:

1. Comparative evaluation of hepatic sinusoidal obstruction syndrome (“HSOS”, according to Jones et al.).
2. Comparative evaluation of donor-type chimerism on Day +28, Day +100, and 12 months after HSCT.
3. Comparative evaluation of OS until 12 months after HSCT.
4. Comparative evaluation of primary and secondary graft failure until 12 months after HSCT.
5. Comparative evaluation of incidence and severity of aGVHD (until Day +100) and cGVHD (until 12 months after HSCT).
6. Comparative evaluation of use of rescue therapies including donor-lymphocyte infusions (DLIs), stem cell infusions with or without further conditioning regimens, re-occurrence of transfusion dependence (ie, necessity of regular transfusions of red blood cells or PLTs).
7. Evaluation of PK parameters of treosulfan and its epoxides and to develop a PK model for assessing relevant covariates.

## Definitions of Exploratory Comparative Analyses

Comparative exploratory analyses included the following and are defined as following:

- Engraftment after transplantation, defined as the first of 3 consecutive days for absolute neutrophil count (ANC) of > 0.5 × 10^9^/L, a leucocyte count of > 1 × 10^9^/L, or platelet (PLT) count of ≥ 20 × 10^9^/L in the absence of platelet transfusion.
- Primary graft failure was diagnosed if an ANC of > 0.5 × 10^9^/L was not achieved after transplantation.
- Complete donor-type chimerism was defined as a donor percentage in bone marrow or peripheral blood of ≥ 95%.
- Mixed donor-type chimerism was defined as a donor percentage of ≥ 20%.
- Transplantation-related mortality (TRM) was defined as the time from end of HSCT to death due to transplantation-related cause.
- Overall survival was defined as the time from end of HSCT to death.
- Graft versus host disease (GVHD) was documented as acute GVHD (aGVHD) or chronic GVHD (cGVHD). Acute GVHD was graded according to the consensus conference on aGVHD grading, cGVHD according to the National Institutes of Health (NIH) criteria for cGVHD.
- GVHD-free survival was defined as the time from end of HSCT to aGVHD of at least Grade III, moderate or severe cGVHD, or death (whichever occurred first).

## Supplemental Information for Parenteral High Dose Administration of Treosulfan^[[1]](#footnote-1)^ Treatment in Practice

Drug name:

L-threitol-1,4-bis-methanesulfonate, treosulfan, Trecondi^®^, Trecondyv^®^

Dose:

Treosulfan dose for children is adapted according to the individual body surface area and 10, 12, or 14 g/m² are administered on three consecutive days. Dose modifications of treosulfan for BMI, hematologic parameters, mild or moderate liver or renal impairment, or other factors are not recommended.

Route:

Treosulfan for conditioning treatment should be infused by central venous access.

Type and volume of diluent:

Dissolve treosulfan (Trecondi^®^) with 0.45% Sodium Chloride Injection or Treosulfan is dissolved in its original glass container by shaking with solvent. Reconstituted solutions may be combined into a larger glass vial, EVA bag or PE bag. Treosulfan 1 g per vial or 5 g per vial are to be reconstituted in 20 mL or 100 mL solvent, respectively. Reconstituted solution must not be stored in the refrigerator but at room temperature. The total treosulfan dose should be infused IV for two hours.

Cycle length and number of cycles:

Conditioning treatment with treosulfan in children is to be administered daily on 3 consecutive days (Day -6, -5, and -4) prior to allogeneic HSCT on Day 0.

Premedications and concurrent medications:

Medications like antibiotics, hydration, antiemetics, growth factors and others are to be administered according to local center standards. Fludarabine (30 mg/m² BSA per day as 0.5 hours IV infusion) is administered on 5 consecutive days (Days ‑6, ‑5, ‑4, ‑3, ‑2) before stem cell infusion (Day 0). The optional additional thiotepa doses (2 × 5 mg/kg on Day -2) are infused over one hour each.

Patient monitoring parameters:

Due to the myeloablative potential of the treosulfan-based conditioning treatment, regular blood tests during treatment until engraftment are mandatory. Healthcare professionals will also do regular blood tests to monitor liver and heart function and to check for other side effects according to local standards.

## Inclusion and Exclusion criteria according to the clinical trial protocol

Inclusion Criteria:

1. Non-malignant disease indicated for first myeloablative allogeneic HSCT, including inborn errors of metabolism, primary immunodeficiencies, haemoglobinopathies and bone marrow failure syndromes.

2. First allogeneic HSCT.

3. Available matched sibling donor (MSD), matched family donor (MFD) or matched unrelated donor (MUD). For bone marrow (BM) and peripheral blood (PB) match is defined as at least 9/10 allele matches after four digit typing in human leucocyte antigen (HLA)-A, -B, -C, –DRB1 and -DQB1 antigens. For umbilical cord blood (UCB) match is defined as at least 5/6 matches after two digit typing in HLA-A and -B and four digit typing in-DRB1 antigens.

4. Age at time of registration from 28 days to less than 18 years of age.

5. Lansky (patients < 16 years of age) or Karnofsky (patients ≥ 16 years of age) performance score of at least 70%.

6. Written informed consent of the parents/legal guardians and patient’s assent/consent according to national regulations.

7. Female patients of child-bearing potential or partner of male patients with child-bearing potential must use a highly effective method of contraception (pearl index < 1%) such as complete sexual abstinence, combined oral contraceptive, hormone intrauterine contraceptive device (IUCD), vaginal hormone ring, transdermal contraceptive patch, contraceptive implant or depot contraceptive injection in combination with a second method of contraception like a condom or a cervical cap / diaphragm with spermicide or surgical sterilisation (vasectomy) in male patients or male partners during the study and at least six months thereafter. For female patients on the study, the vasectomised male partner should be the sole partner for that patient.

8. Negative pregnancy test for females of child-bearing potential.

Exclusion Criteria:

1. Second or later HSCT.

2. HSCT from mismatched donor (less than 9/10 BM/peripheral blood stem cells (PBSC) or less than 5/6 matched cord donor).

3. Preterm newborn infants (< 37 weeks gestational age) and term newborn infants aged 0–27 days at time of registration.

4. Obese paediatric patients with body mass index weight (kg)/[height (m)]² > 30 kg/m².

5. Diagnosis of Fanconi anaemia and other chromosomal breakage disorders, radiosensitivity disorders (deoxyribonucleic acid (DNA) Ligase 4, Cernunnos- X-ray repair cross-complementing protein 4 (XRCC4) like factor (XLF), Nijmegen Breakage Syndrome (NBS)) and Dyskeratosis Congenita.

6. Treatment with cytotoxic drugs within 10 days prior to day -7.

7. Impaired liver function indicated by Bilirubin > three times the upper limit of normal (ULN) or aspartate aminotransferase/alanine aminotransferase (AST/GOT, ALT/GPT) > ten times ULN, or clinically significant coagulopathy, or active infectious hepatitis with clinical evidence.

8. Impaired renal function indicated by estimated glomerular filtration rate ([GFR], according to the Schwartz formula) < 60 mL/min/1.73m2.

9. Impaired cardiac function: severe cardiac insufficiency indicated by left ventricular ejection fraction (LVEF) ≤ 35%.

10. Requirement for supplementary continuous oxygen.

11. Severe active infection requiring deferral of conditioning.

12. Human immunodeficiency virus (HIV) positivity.

13. Severe concomitant illness, comorbidity or condition that would severely limit life expectancy.

14. Known pregnancy, breast feeding.

15. Known hypersensitivity to Treosulfan, Busulfan,

Fludarabine and/or Thiotepa.

16. Participation in another interventional clinical study with an experimental drug, within four weeks prior to patient inclusion.

# Supplemental Tables

Supplemental Table 1: Frequency of Subjects with Treatment-emergent Adverse Events of at least CTCAE Grade III by System Organ Class and Preferred Term Occurring in at least 10% of Subjects in Either Treatment Arm

| **System Organ Class**  **Preferred Term** | **Busulfan**  **(N=50)** | **Treosulfan**  **(N=51)** |
| --- | --- | --- |
| Subjects with any event | 41 (82.0%) | 41 (80.4%) |
| **Gastrointestinal disorders** |  |  |
| Mucositis oral | 24 (48.0%) | 14 (27.5%) |
| Diarrhea | 7 (14.0%) | 7 (13.7%) |
| Nausea | 9 (18.0%) | 3 (5.9%) |
| Vomiting | 7 (14.0%) | 4 (7.8%) |
| **Infections and infestations** |  |  |
| Infections and infestations - Other, specify | 4 (8.0%) | 14 (27.5%) |
| Sepsis | 5 (10.0%) | 3 (5.9%) |
| Lung infection | 5 (10.0%) | 1 (2.0%) |
| **Vascular disorders** |  |  |
| Hypertension | 6 (12.0%) | 8 (15.7%) |

CTCAE, Common Terminology Criteria for Adverse Events; N, total number of subjects.

Supplemental Table 2: Summary of Detailed Causes of Deaths

|  | **Busulfan**  **(N=50)** | **Treosulfan**  **(N=51)** |
| --- | --- | --- |
| **Survival status at trial termination, n (%)** |  |  |
| Alive* | 43 (86.0%) | 49 (96.1%) |
| Dead | 7 (14.0%) | 2 (3.9%) |
| **If dead, cause of death^†^, n (%)** |  |  |
| Transplantation related^‡^ | 7 (14.0%) | 2 (3.9%) |
| GVHD | 2 (4.0%) | 2 (3.9%) |
| Pulmonary toxicity | 1 (2.0%) | 0 (0.0%) |
| Hemorrhage | 1 (2.0%) | 0 (0.0%) |
| Renal failure | 0 (0.0%) | 1 (2.0%) |
| Multiple organ failure | 1 (2.0%) | 2 (3.9%) |
| Infection | 5 (10.0%) | 1 (2.0%) |
| Bacterial | 2 (4.0%) | 1 (2.0%) |
| Viral | 2 (4.0%) | 0 (0.0%) |
| Fungal | 1 (2.0%) | 0 (0.0%) |
| Unspecified | 0 (0.0%) | 1 (2.0%) |
| Interstitial pneumonitis | 1 (2.0%) | 0 (0.0%) |
| EBV proliferative disease | 0 (0.0%) | 1 (2.0%) |
| Gastrointestinal toxicity | 0 (0.0%) | 1 (2.0%) |
| Other transplant related cause | 1 (2.0%) | 0 (0.0%) |

* The status ‘alive’ is displayed for all subjects who did not terminate the trial due to death.

† Causes answered 'Unknown' are not counted.

‡ Multiple transplantation related causes per subject possible.

EBV, Epstein-Barr virus; GVHD, graft-versus-host disease; N, total number of subjects; n, number of subjects in each category.

Supplemental Table 3: All Patients with Graft Failure and Their Survival Status

| **Serial number** | **Diagnosis** | **Source stem cells / donor type** | **Treatment** | **Graft failure** | **Time to graft failure (days since HSCT)** | **Last visit** | **Survival status** | **Serotherapy (Yes/No)** | **Last chimerism at time of graft failure (%)** |
| --- | --- | --- | --- | --- | --- | --- | --- | --- | --- |
| 1 | Mucopolysaccaridoses | CB / MFD | Busulfan | Primary | 21 | Month 48 | Alive | No | ND |
| 2 | Mucopolysaccaridoses | CB / MUD | Busulfan | Primary | 27 | Day 28 | TRM (98 days after HSCT) | No | ND |
| 3 | CD 40 ligand deficiency | PB / MUD | Treosulfan | Secondary | 342 | Month 60 | Alive | Yes | ND |
| 4 | Bone marrow failure with single cytopenia (Congenital neutropenia) | BM / MUD | Treosulfan | Secondary | 369 | Month 24 | Alive | Yes | 25.7 |
| 5 | Beta thalassaemia major | PB / MUD | Treosulfan | Secondary | 371 | Month 12 | Alive | Yes | 5 |
| 6 | Sickle cell disease | PB / MUD | Treosulfan | Secondary | 260 | Month 24 | Alive | Yes | 20 |
| 7 | IPEX, FOXP3 mutation | CB /MUD | Treosulfan | Primary | 28 | Month 48 | Alive | Yes | 0 |
| 8 | Bone marrow failure with single cytopenia (Blackfan-Diamond Anaemia) | BM / MUD | Treosulfan | Secondary | 266 | Month 24 | Alive | Yes | 2 |
| 9 | Chronic granulomatous disease | BM / MUD | Treosulfan | Secondary | 183 | Month 48 | Alive | Yes | 12 |
| 10 | Chronic granulomatous disease | BM / MUD | Treosulfan | Secondary | 88 | Month 48 | Alive | Yes | 20 |
| 11 | Beta thalassaemia major | BM / MUD | Treosulfan | Secondary | 743 | Month 24 | Alive | Yes | 4.66 |
| 12 | Beta thalassaemia major | BM / MUD | Treosulfan | Primary | 54 | Month 24 | Alive | No | 2 |
| 13 | Pure red cell aplasia | BM / MUD | Treosulfan | Secondary | 26 | Month 24 | Alive | No | 0 |

BM, bone marrow; FOXP3, forkhead box protein 3; HSCT, hematopoietic stem cell transplantation; IPEX, immune dysregulation, polyendocrinopathy, enteropathy, X-linked; MFD, matched family donor; MUD, matched unrelated donor; ND, not done; PB, peripheral blood; TRM, transplantation-related mortality; CB, cord blood.

# Supplemental Figures

Supplemental Figure 1: Kaplan-Meier Estimates of GVHD-free Survival


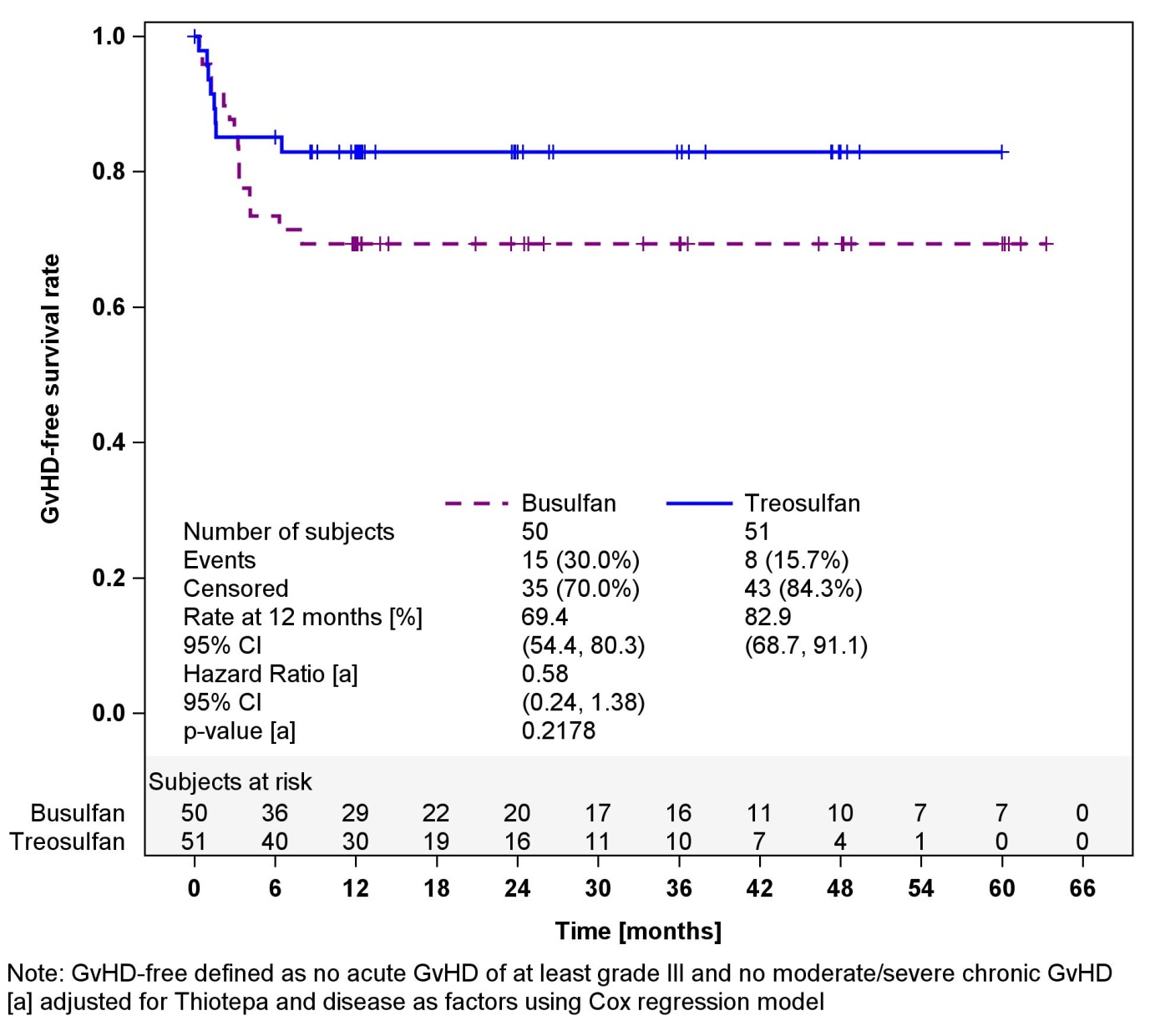


Supplemental Figure 2: Kaplan-Meier Estimates of Chronic GvHD-free Survival


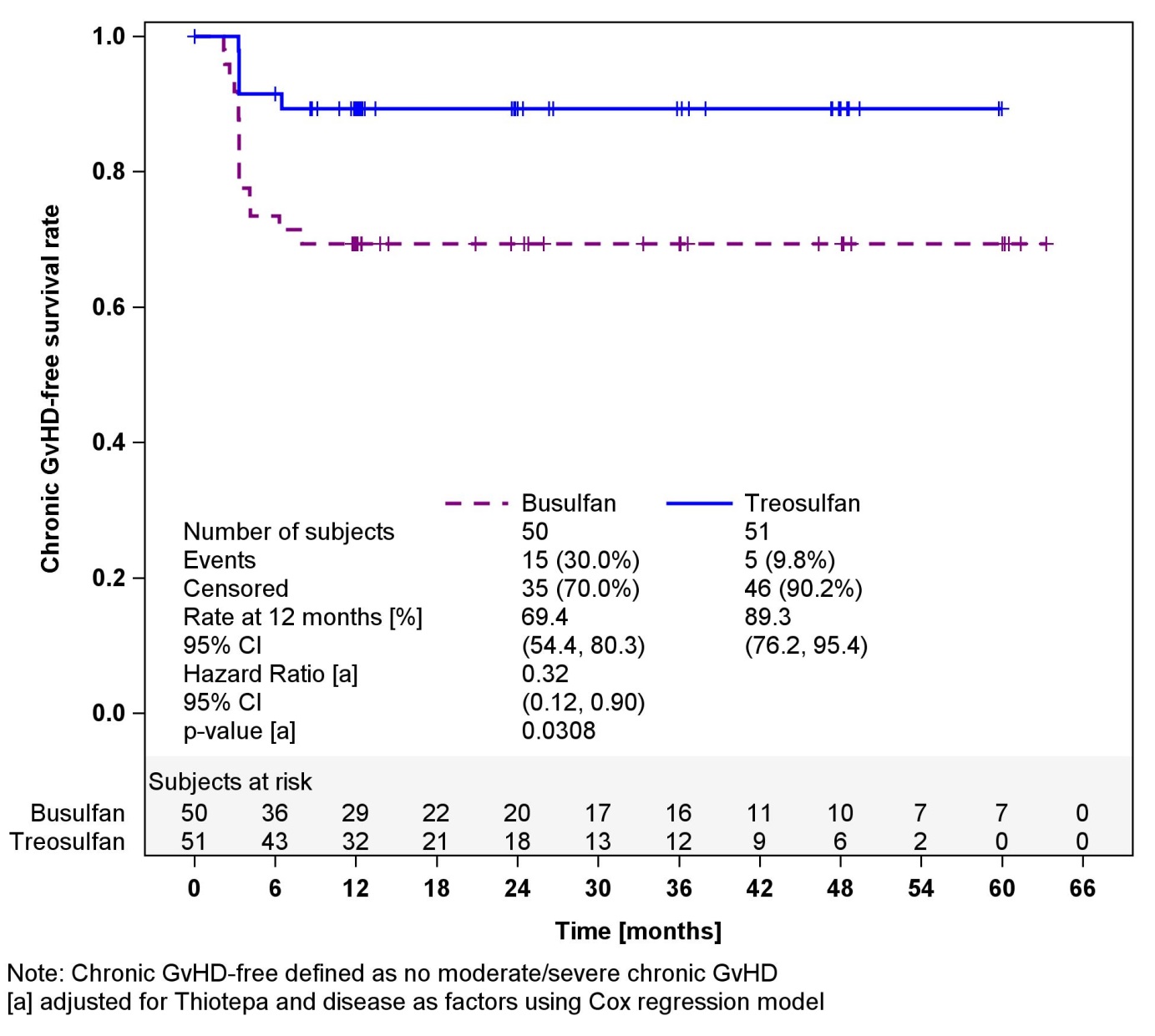


Supplemental Figure 3: Forest Plot for Freedom from Transplantation (Treatment)-related Mortality Until Day +100 By Subgroups

**
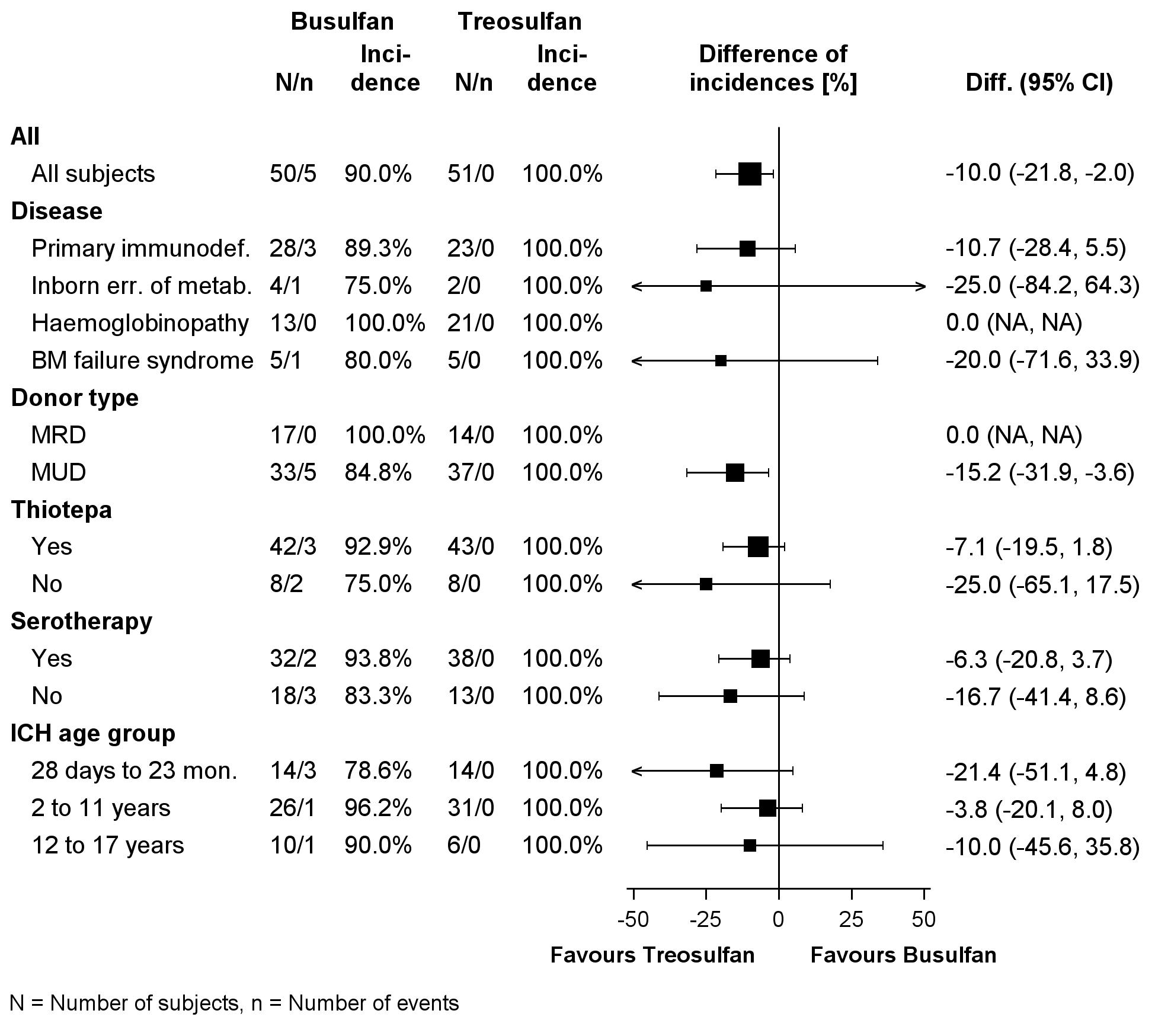
**

Supplemental Figure 4: Cumulative Incidence of Graft Failure


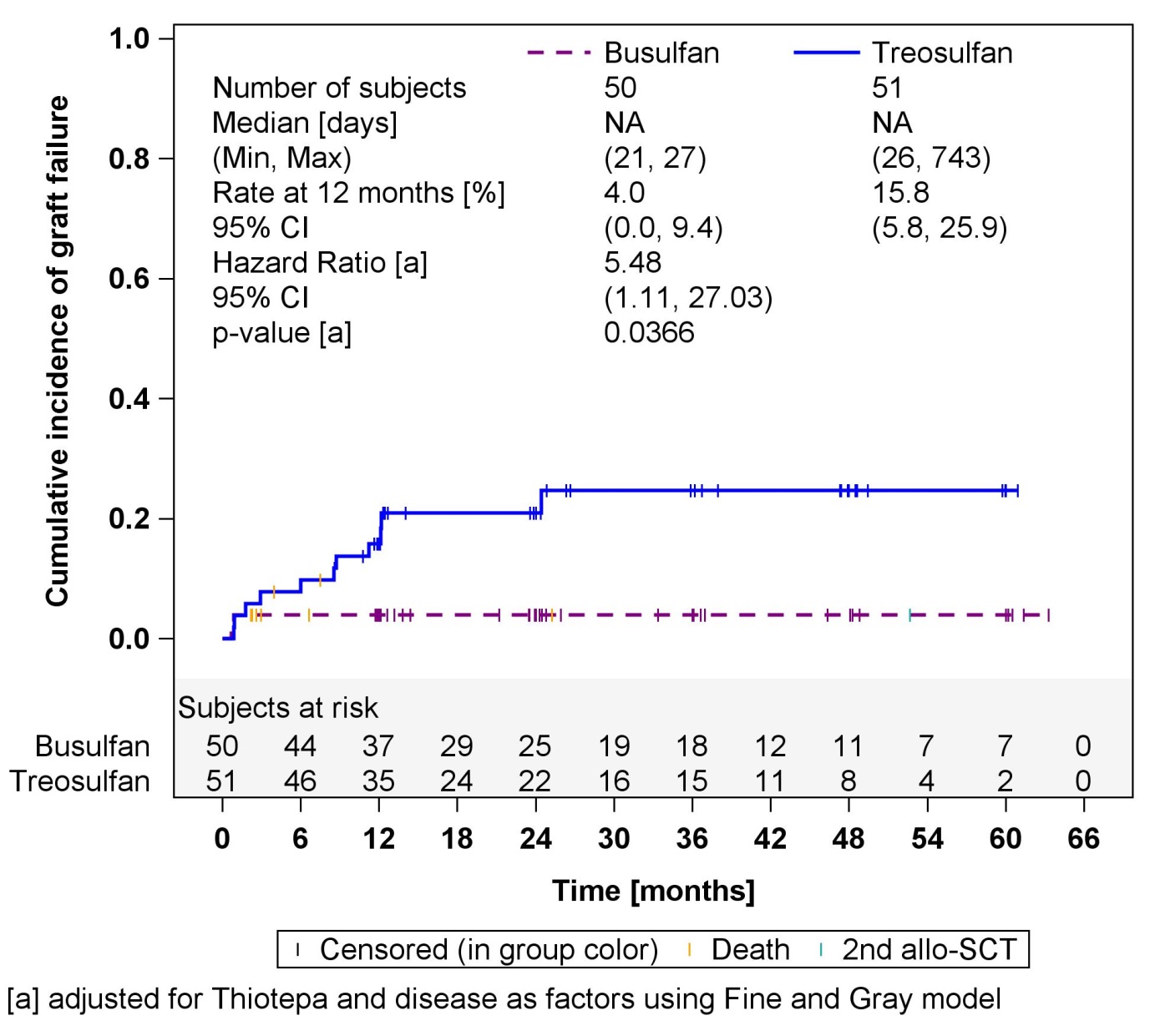


1. Intravenous busulfan-based (Busilvex^®^, Pierre Fabre Medicament) conditioning treatment was administered in accordance with European Medicines Agency approved Summary of Product Characteristics. [↑](#footnote-ref-1)
